# Meeting Report: Risk Assessment of Tamiflu Use Under Pandemic Conditions

**DOI:** 10.1289/ehp.11310

**Published:** 2008-05-30

**Authors:** Andrew C. Singer, Bruce M. Howard, Andrew C. Johnson, Chris J. Knowles, Simon Jackman, Cesare Accinelli, Anna Barra Caracciolo, Ian Bernard, Stephen Bird, Tatiana Boucard, Alistair Boxall, Jayne V. Brian, Elise Cartmell, Chris Chubb, John Churchley, Sandra Costigan, Mark Crane, Michael J. Dempsey, Bob Dorrington, Brian Ellor, Jerker Fick, John Holmes, Tom Hutchinson, Franz Karcher, Samuel L. Kelleher, Peter Marsden, Gerald Noone, Miles A. Nunn, John Oxford, Tony Rachwal, Noel Roberts, Mike Roberts, Maria Ludovica Saccà, Matthew Sanders, Jürg Oliver Straub, Adrian Terry, Dean Thomas, Stephen Toovey, Rodney Townsend, Nikolaos Voulvoulis, Chris Watts

**Affiliations:** 1 Centre for Ecology & Hydrology, Oxford, United Kingdom; 2 Environmental Knowledge Transfer Network, University of Oxford, Oxford, United Kingdom; 3 Centre for Ecology & Hydrology, Wallingford, Oxfordshire, United Kingdom; 4 University of Bologna, Bologna, Italy; 5 Italian National Research Council, Rome, Italy; 6 South West Water, Exeter, United Kingdom; 7 Environment Agency, Wallingford, United Kingdom; 8 University of York, York, United Kingdom; 9 Brunel University, London, United Kingdom; 10 Cranfield University, Cranfield, United Kingdom; 11 Environment Agency, Bristol, United Kingdom; 12 Severn Trent Water, Coventry, United Kingdom; 13 UK Government Department of Health, London, United Kingdom; 14 WCA Environment Ltd., Faringdon, United Kingdom; 15 Manchester Metropolitan University, Manchester, United Kingdom; 16 Northumbrian Water Group (Essex & Suffolk Water), Chelmsford, United Kingdom; 17 United Utilities, Warrington, United Kingdom; 18 Umeå University, Umeå, Sweden; 19 University of Oxford, Oxford, United Kingdom; 20 AstraZeneca, Brixham, Devon, United Kingdom; 21 Health Threats Unit, European Commission, Luxembourg; 22 Drinking Water Inspectorate, London, United Kingdom; 23 Worshipful Company of Water Conservators, London, United Kingdom; 24 Retroscreen Ltd./Barts and The London, Queen Mary’s School of Medicine and Dentistry, London, United Kingdom; 25 Independent Consultant, West Molesey, Surrey, United Kingdom; 26 F. Hoffmann-La Roche Ltd, Basel, Switzerland; 27 UK Government Department for Environment, Food and Rural Affairs, London, United Kingdom; 28 Centre for Environment, Fisheries and Aquaculture Science, Weymouth, United Kingdom; 29 Cambridge Environmental Assessments, Cambridge, United Kingdom; 30 Royal Society of Chemistry, London, United Kingdom; 31 Imperial College London, London, United Kingdom

**Keywords:** antiviral, drug, ecotoxicology, influenza, pandemic, pharmaceutical, pollution, sewage treatment plant, Tamiflu

## Abstract

On 3 October 2007, 40 participants with diverse expertise attended the workshop Tamiflu and the Environment: Implications of Use under Pandemic Conditions to assess the potential human health impact and environmental hazards associated with use of Tamiflu during an influenza pandemic. Based on the identification and risk-ranking of knowledge gaps, the consensus was that oseltamivir ethylester-phosphate (OE-P) and oseltamivir carboxylate (OC) were unlikely to pose an ecotoxicologic hazard to freshwater organisms. OC in river water might hasten the generation of OC-resistance in wildfowl, but this possibility seems less likely than the potential disruption that could be posed by OC and other pharmaceuticals to the operation of sewage treatment plants. The work-group members agreed on the following research priorities: *a*) available data on the ecotoxicology of OE-P and OC should be published; *b*) risk should be assessed for OC-contaminated river water generating OC-resistant viruses in wildfowl; *c*) sewage treatment plant functioning due to microbial inhibition by neuraminidase inhibitors and other antimicrobials used during a pandemic should be investigated; and *d*) realistic worst-case exposure scenarios should be developed. Additional modeling would be useful to identify localized areas within river catchments that might be prone to high pharmaceutical concentrations in sewage treatment plant effluent. Ongoing seasonal use of Tamiflu in Japan offers opportunities for researchers to assess how much OC enters and persists in the aquatic environment.

Under the guidance of the World Health Organization (WHO), 41 nations have developed pandemic preparedness plans describing the role different organizations will play when confronted with an influenza pandemic [European Influenza Surveillance Scheme (EISS) 2007; Mounier-Jack et al. 2007]. The plans aim to maintain essential services, reduce disease transmission and the socioeconomic consequences of a pandemic, and minimize the number of infectious cases, hospitalizations, and deaths (EISS 2007; Mounier-Jack et al. 2007).

The WHO has strongly recommended the use of the antiviral Tamiflu, produced and distributed by F. Hoffmann-La Roche Ltd. (Basel, Switzerland), as the primary choice for combating an influenza pandemic (WHO 2006a). Tamiflu was recommended because *a*) there is low natural viral resistance (Aoki et al. 2007; Roberts 2001); *b*) it is easy to administer orally via capsule; *c*) it is systemically active; and *d*) it is effective against characterized influenza A and B viruses (Ward et al. 2005; WHO 2006b). International stockpiles of influenza A antivirals have been growing rapidly since 2005 (Figure 1), and most countries are stockpiling sufficient quantities of antiviral to treat 25% of their population (Department of Health and Human Services 2006; Ferguson et al. 2006; Roche 2007). Stockpiles are anticipated to continue to increase toward a 50% coverage goal in some countries. In addition, stockpiles are likely to diversify, incorporating additional neuraminidase inhibitors (NAI) such as zanamivir (Relenza; GlaxoSmithKline, London, UK) (Ferraris et al. 2005) and peramivir (Biocryst Pharmaceuticals, Cary, NC, USA) (Smee and Sidwell 2002), as well as traditional antivirals such as amantadine and rimantadine (WHO 2007).

The United Kingdom has stockpiled 14.6 million courses of Tamiflu, equating to nearly 11 metric tons of oseltamivir ethylester-phosphate (OE-P), all of which is expected to be used for treatment during the 9- to 12-week period of a pandemic. OE-P use has been identified as a potentially unacceptable risk, and various potential effects and exposures are associated with oseltamivir carboxylate (OC) (Singer et al. 2007). The following criteria have been used to assess the risks posed by OE-P use during an influenza pandemic:

- Renal and fecal excretion of the oral dose of OE-P is in its active antiviral form OC (F. Hoffmann-La Roche 2007)
- Negligible biotransformation of OC in sewage treatment plants (STPs) [European Medicines Agency (EMEA) 2005; Fick 2007]
- Low sorption of OC into sewage sludge (low Log P) and high water solubility (F. Hoffmann-La Roche 2007)
- Negligible biodegradation of OC in river water (Accinelli et al. 2007)
- Insufficient dilution of OC in many of the examined receiving river waters to obviate ecotoxicologic risks (Singer et al. 2007).

## Preliminary hazard characterization

It has been suggested that the release of OC into rivers generates OC resistance in avian influenza in wildfowl (Singer et al. 2007). OC could enter the gut of wildfowl from ingested river water and interact with the avian influenza neuraminidase. The concentration of OC in the gut might be higher than in river water owing to recycling of the urine in waterfowl, thereby further increasing the selection pressure for OC resistance.

The workshop, Tamiflu and the Environment: Implications of Use under Pandemic Conditions, was designed to further characterize the hazards and risks associated with the projected scale of Tamiflu release to the environment during a pandemic, as well as to identify priorities for further research. It was recognized by the workshop’s organizers that a holistic assessment of risks could be made best by bringing together diverse experts and organizations with relevant experience. Accordingly, experts in environmental chemistry, ecotoxicology, virology, microbiology, enzymology, hydrology, public health protection, and wastewater engineering were invited to participate. The pharmaceutical industry, the water industry, and central government were represented, together with a wide variety of organizations advising and supporting these sectors.

## Overview

To ensure that all participants had the knowledge base required, plenary talks provided an overview of the current understanding of the processes and hazards associated with OC release to the environment.

In selected U.S. and U.K. catchments during an influenza pandemic, the predicted environmental concentration (PEC) of OC depends on population size and liters of river water available for dilution of sewage effluent per capita. Concentrations of OC in catchments with particularly low flow and high populations are predicted to be > 20 μg/L, which is significantly higher than that observed for most other pharmaceutical contaminants. OC may possibly affect the function and stability of sewage treatment plants (STPs) as a result of the inhibition of floc or biofilm formation, as these microbial growth forms are integral to process stability and functionality. Because a mixture of pharmaceuticals—particularly antibiotics—is likely to pass through STPs during a pandemic, the risk of inhibition of floc or biofilm formation could be even greater. Also, the generation of OC resistance in avian influenza–infected wildfowl after exposure to OC-contaminated river water would be difficult to manage because of the migratory nature of the hosts.

Further pandemics could be avoided with a concerted research effort including robust preparedness plans, especially the use of antivirals. The proportion of the population receiving Tamiflu could be higher than the 25% considered by Singer et al. (2007). Although it is inevitable that drug-resistant forms of the influenza virus will occur, strains of influenza that are resistant to pharmaceuticals have been found to have compromised biological fitness (Aoki et al. 2007).

The principles of environmental assessment processes have been reported by the EMEA (2006). Regarding the current regulatory framework for predicted no-effect concentration (PNEC) and PEC assessments for human pharmaceuticals in various countries, Europe emphasizes chronic effects assessment in algae, crustaceans, and fish, whereas the United States focuses on the assessment of acute effects. The European Centre for Ecotoxicology & Toxicology of Chemicals (ECETOC 2007) argues for intelligent strategies for chronic ecotoxicity testing that reflects the mode-of-action protein target(s) of a given chemical (e.g., agrochemical, biocide, pharmaceutical). For example, chronic testing of estrogenic drugs should include aquatic animal species that are known to have estrogen receptors. Wider use of the mode-of-action intelligent testing strategy approach as an alternative to routine lethality testing would provide important animal welfare and economic benefits while simultaneously providing a sound scientific rationale for calculating PNECs for OE-P, OC, and other important human pharmaceuticals.

Unpublished results from ongoing Organisation for Economic Co-operation and Development (OECD) environmental degradation and toxicity tests on environmentally and physiologically relevant mixtures of OE-P and OC were presented. The available data indicate that most of the dose received by the human population will pass through sewage treatment works and therefore enter surface waters, with negligible removal from the water column to sediments. Chronic ecotoxicity testing has been conducted in light of the 2006 EMEA guidelines on environmental risk assessment for human pharmaceuticals requiring PNECs based on chronic data (EMEA 2006) and the projected ≥ 8 weeks of OC release into receiving rivers during a pandemic (Singer et al. 2007). These chronic ecotoxicity tests were performed with green algae *(Pseudokirchneriella subcapitata*) in a growth inhibition test, with *Daphnia magna* in a reproductive toxicity study, and with zebrafish in an early-life-stage test, all following OECD guidelines 201 (OECD 1984), 211 (OECD 1998), and 210 (OECD 1992), respectively, and performed under Good Laboratory Practice quality assurance. The preliminary no observed effects concentrations (NOECs) resulted in a PNEC of 100 μg/L, applying an assessment factor of 10. This PNEC is higher than published PECs (Singer et al. 2007) or those newly calculated using worst-case pandemic use assumptions and various algorithms. Hence, based on recognized environmental risk assessment procedures as detailed in the European Union *Technical Guidance Document on Risk Assessment* (European Commission 2003), risk from OE-P and OC in the scenarios presented appear to be negligible, including the low-dilution scenario in the River Lee in the United Kingdom (Singer et al. 2007).

The U.K. Environment Agency does not have any role in the licensing of human pharmaceuticals or the environmental safety assessments required by the regulatory process. In the United Kingdom the responsibility for issuing licenses lies with the Medicines and Healthcare products Regulatory Agency (MHRA). Furthermore, the Environment Agency has no advisory role in this process. By contrast, the Environment Agency acts as an advisor to the relevant competent authorities for pesticides, biocides, and veterinary pharmaceuticals for issues relating to environmental safety. The program of work on human pharmaceuticals lies within the Environment Agency’s responsibility for assessing and reporting on the state of the environment, as well as identifying possible environmental concerns. This work includes a screening process used to rank pharmaceuticals based on their relative risk to the aquatic environment (Environment Agency 2003, 2008) and a short, targeted monitoring program conducted for a number of the higher-priority pharmaceuticals (Environment Agency 2003). OE-P was not included in the screening process because of its low usage in the United Kingdom for routine treatment. The Environment Agency therefore conducted a separate assessment for use under pandemic conditions, drawing on public information sources. Exposure was estimated for treatment only and for treatment plus prophylaxis, using assumptions from Singer et al. (2007) and modified assumptions from the Department of Health based on treatment of 50% of the population with Tamiflu and prophylaxis (Scientific Pandemic Influenza Advisory Committe 2008). Based on available data, risk to the aquatic environment from OE-P and OC appeared low. However, this requires further investigation for catchments with high population and low dilution of sewage effluents in surface waters. The Environment Agency will review new data generated on fate and effects of OC (e.g., by F. Hoffmann-La Roche) before reaching any further conclusions (T. Boucard, personal communication).

River water pollution with pharmaceuticals is relevant in the United Kingdom, particularly in England, because it is a densely populated, small island with relatively short low-flow rivers (Keller et al. 2006). More precise modeling is needed to determine specific locations where local risks to water pollution are greatest. Hydrologic and demographic factors in the United Kingdom indicate that the Midlands, Thames, and Anglian regions of England are likely at highest risk.

## Aim and Objectives

The aim of the workshop was to assess the implications of Tamiflu release to the environment following mass administration under pandemic conditions, and to identify any further actions required to minimize risks to human and environmental health.

Four multidisciplinary working groups addressed the following questions:

1. Does current knowledge about Tamiflu release to the environment provide sufficient assurance of safety for human health and the environment?
2. What are the research needs to ensure that the risks associated with Tamiflu release to the environment can be better understood, minimized, or mitigated?
3. Of any research requirements identified, what are the priority research tasks?
4. What are the long-term issues triggered by, or associated with, the issue of Tamiflu release to the environment?

## Results

Workgroup members were asked to quantify their assurance of safety with the present knowledge base on Tamiflu release to the environment on a scale of 1–5 (where 1 = low assurance of safety and 5 = high assurance of safety). The mean ± SD rating for the four working group sessions was 3.3 ± 0.3.

The highest priority knowledge gaps identified by participants fell into four main areas: *a*) ecotoxicologic effects; *b*) antiviral resistance; *c*) STP failure, particularly as a result of nontarget neuraminidase inhibition (e.g., microorganisms); and *d*) exposure models to define realistic worst-case scenarios for environmental exposure.

### Ecotoxicity

Most participants concluded that the ecotoxicity of OE-P and OC was not likely to be an area of primary concern based on preliminary data (Straub JO, personal communication). Many participants expressed a desire to see a broader range of ecotoxicologic work conducted and published in peer-reviewed journals. However, the level of concern regarding the ecotoxicity was generally quite low. Considerably greater concern was expressed regarding the potential inhibition of nontarget neuraminidases in organisms other than influenza viruses (e.g., microorganisms).

### Antiviral resistance

Workshop participants were uncertain about the potential for generation of OC resistance in avian influenza viruses as a result of the exposure of wildfowl to OC in surface waters. Concerns were somewhat alleviated by the knowledge that OC is not readily absorbed from the gut and therefore should not be present in significant amounts in the urine to recycle, as proposed by Singer et al. (2007). The consensus opinion was that it is difficult to predict the exposure of OC in the wild fowl gut and its implications for hastening the generation of OC-resistance in avian influenza. However, attending virologists agreed that reassortment, or mutation, would be required to generate a virus with human specificity, and such viruses would have to find a path to circulate back to humans. The actual risk to the human population is unlikely to be significant. Nevertheless, the possibility of OC resistance arising in influenza viruses in waterfowl, although unlikely, cannot be categorically ruled out. Thus, it is important to consider the consequences if resistant viruses should evolve (be selected).

The highest concentrations of OC in river water will be reached only during relatively short spells of very high drug usage during an influenza pandemic. This can occur only after thousands of infected patients have been treated with the drug. Resistant viruses will inevitably evolve in some of these patients in advance of any possible selection in waterfowl. Thus, should the waterfowl be infected with the pandemic strain, the resistant virus strains and genetic mutations encoding OC resistance selected will be common to both species but selected in humans first. A resistant pandemic virus in waterfowl, alongside the same viruses already in humans, will have little or no effect on the human situation. Similar to the situation in humans, resistant viruses in waterfowl are likely to be cleared or revert to wild-type once river OC levels fall and the selection pressure is removed. Available data regarding the nature of OC resistance indicate that mutations responsible for conferring resistance severely compromise viral growth and infectivity (Aoki et al. 2007; Chutinimitkul et al. 2007; Hayden 2006; Hurt et al. 2007; Lipsitch et al. 2007; Yen et al. 2005). If resistance were selected or acquired by reassortment in other avian virus strains, again the resistant viruses would likely revert to wild-type once selection pressure was removed. If for some reason this did not happen and the resistant virus persisted as one of the avian virus strains circulating in waterfowl, then its chance of becoming a human pathogen (by chance mutation or recombination) would be no greater than that for any of the other 16 avian influenza A virus serotypes in circulation. Such events are rare; highly pathogenic H5N1 viruses have been circulating and infecting the occasional human who has direct contact with birds since 1997, and the virus has still not achieved the capability of transmitting between humans. Thus, the idea of a “second wave” pandemic arising by this mechanism is considered highly unlikely.

There is concern that nonpandemic strains might not be a good predictor of how a pandemic strain of influenza could develop, because the possibility of developing compensatory mutations could allow an OC-resistant pandemic strain to grow and infect in an uncompromised manner. There appears to be a gap between the perceptions of nonvirologists working in this field and the views expressed by virologists that emergence of resistance is unlikely to pose a threat.

### STP failure

In the event of an influenza pandemic, STPs will receive approximately 10-fold higher concentrations of OC (and other antiviral or antimicrobial compounds) than is projected to occur in rivers (Singer et al. 2007), because the STP effluent is diluted by river flow. This higher concentrations will pose a widespread problem to STPs if these compounds inhibit the process organisms during a pandemic. One of the concerns is that OC might exhibit activity on neuraminidases produced by bacteria within STPs. Because there are potential analogous receptors that might respond to the release of Tamiflu, it would be prudent to assess the nontarget “activity” of the drug. Tamiflu is an NAI that was rationally designed to inhibit the influenza A and B neuraminidase; however, there is a basis for considering that a neuraminidase present in a bacterium could also be inhibited by Tamiflu. Soong et al. (2006) demonstrated the efficacy of OC and a similar antiviral, peramivir, to inhibit biofilm formation in the microorganism *Pseudomonas aeruginosa*. These authors were interested in the potential to use the NAI for alleviating symptoms of cystic fibrosis, hence they did not investigate environmentally relevant concentrations of OC. Nevertheless, they found that 1 μg/L OC demonstrated a 0.6-fold inhibition of biofilm formation, which might be applicable to concentrations in STPs during an influenza pandemic (Soong et al. 2006). This demonstration of the importance of bacterial neuraminidases in biofilm formation indicates the possible vulnerability of STP biofilms to OC exposure during a pandemic. There are additional concerns regarding STP failure because of the potential scale of antibiotic use to treat secondary infections stemming from influenza-infected patients, although the use of antivirals might decrease the need for antibiotic use (Kaiser et al. 2003; Nicholson et al. 2000; Treanor et al. 2000; Whitley et al. 2000).

Changes in activated sludge floc integrity or the biofilms in trickling filter works could lead to a loss of effective sewage treatment. This would have catastrophic environmental consequences, as untreated sewage entering rivers would kill a large number of the aquatic organisms and be an additional threat to human health. Another concern is that restarting sewage works with fresh cultures is a difficult process.

### Exposure models

One example of routine use of OE-P for seasonal influenza is Japan, which maintains the highest use of NAIs of any country in the world. Greater than 90% of the prescriptions for seasonal influenza in Japan are for OE-P (Moscona and McKimm-Breschkin 2007). Sewage works in Japan have been processing OC from 5–10% population treatment per annum for > 4 years. There is concern regarding this use pattern and whether sufficient OC is released into rivers within Japan to generate an OC-resistant strain even before the onset of a pandemic. However, the risk posed by the generation of OC-resistance in the environment from OC-containing sewage effluent is likely to be low, for the aforementioned reasons. Japan might provide an opportunity to test the validity of the model systems used to predict river levels of OC during high drug usage and to examine the long term effect of high OC levels on sewage treatment efficiency and biofilms in real life situations. Swabbing of wildfowl in Japan for OC-resistant virus could be conducted to detect resistance events, but as these are likely to be rare, the results of such a study would be equivocal without very high sample numbers.

## Conclusions

By assembling a wide range of relevant expertise, Tamiflu and the Environment: Implications of Use under Pandemic Conditions provided a unique opportunity to make a preliminary holistic assessment of whether safety for the environment and human health can be assured if Tamiflu is used under pandemic conditions. Although questions of safety assurance depend on professional judgment, the consensus of this workshop was that OE-P and OC release into the environment might still pose risks associated with the generation of antiviral resistance or destabilization of microbial biofilms that are key to the performance and function of STPs. The risk of OC resistance in wildfowl seems to be less significant than the effects of mixtures of pharmaceuticals in sewage and the sewage treatment process. This is a critical issue given the unprecedented quantities of analgesics, anti-inflammatory drugs, antipyretics, antibiotics, and antivirals likely to be used in a pandemic. The case of seasonal use of Tamiflu in Japan might provide a valuable surrogate for assessing the implications of release under pandemic conditions, as well as validating models.

Potential inhibition of microbial neuraminidases raised additional questions pertaining to the pharmaceutical regulatory process: First, should additional tests be performed on “nontarget” organisms (e.g., bacteria) and scenarios (e.g., STPs) based on the mode of action? Second, should the environmental safety of pharmaceuticals be specifically evaluated for pandemic scenarios, where appropriate?

The assessment of pharmaceutical release to the environment, particularly that projected to occur under epidemic or pandemic conditions, requires the integration of a diverse range of scientific expertise across a range of public and private organizations. As part of this, national environmental regulators should play a more active role informing international and national authorities governing pharmaceutical use. Clearly, the implications for the water industry of potentially large quantities of antimicrobial products entering STPs need to be assessed more carefully.

The risk scenarios proposed here are not unique to Tamiflu or to an influenza pandemic, but to all future epidemics and pandemics. Hence, the output of the workshop provides an initial effort toward developing fully integrated preparedness plans that consider all facets of human and environmental health.

## Recommendations

Recommendations from Tamiflu and the Environment: Implications of Use under Pandemic Conditions are as follows:

- The vulnerability of STPs should be examined regarding exposure to the predicted concentrations of OC and antibiotics during a pandemic.
- All newly generated environmental fate and ecotoxicologic data for OC and OE-P, along with an environmental risk assessment, should be collated and published in a peer-reviewed scientific journal.
- More detailed models should be used to assess the highest likely environmental concentrations of OC and associated antimicrobials both in STPs and in catchments known to have low dilution.

## Correction

In the manuscript originally published online, the listing of authors was inaccurate. It has been corrected here.

## Figures and Tables

**Figure 1:**
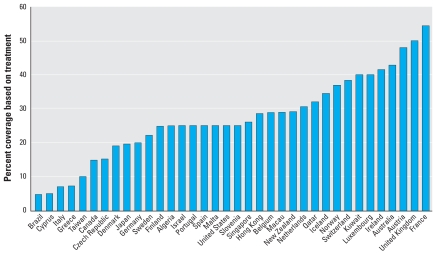
Government Tamiflu targets for population coverage (%). These values include the number of antiviral treatments that governments have stockpiled or intend to stockpile, as a percentage of the total country population. These values are publicly available either via national pandemic plans or media releases; they may not be a true reflection of actual stockpiles (i.e., some governments may have stockpiled more but have not publicly communicated updated figures); include only countries that have or intend to stockpile for > 5% of their population; and include antiviral stockpiles (in some cases the coverage includes Tamiflu and Relenza, and in others only Tamiflu as of November 2007).
